# Turpeth Mineral in Croup

**Published:** 1846-01

**Authors:** 


*Turpeth Mineral.—Croup.* Dr. Hubbard, of Maine, recommends this substance as a prompt, safe, and valuable emetic in certain diseases. We find his remarks in the Summary of the Transactions of the College of Physicians of Philadelphia:

My attention was first directed to the use of this substance from feeling the want of some article, reliable, as an emetic of certain, prompt, efficient, and safe action, with the least tendency to run off by the bowels. For, whatever may be thought of the value of emetico-cathartics, in many other cases, it must be admitted by all, that simple emesis is, in many pathological conditions, alone desirable; and that its complication with catharsis will, in such conditions, not only defeat the beneficial effects of the emesis, but will prove positively injurious, and often extremely hazardous. It is under precisely such conditions of the system, that the emetics, in common use, such as tart. antimon., ipecac., sulph. zinc, &c., will most frequently fail of producing efficient emesis, and are most liable to run off by the bowels. There are certain diseases—for instance, croup, scarlatina maligna—during all the stages of which, there is great insensibility of the stomach to the impression of emetics, and, when produced by inordinate doses, emesis is likely to be very imperfect, and to be followed by catharsis. The same insensibility also exists in all diseases, under circumstances of great prostration of the system, whether that prostration be owing to the violence of the disease or to its duration.

Every practitioner of experience must have felt the importance of availing himself, under some of the above named pathological conditions, of the revellent and equalizing effect of simple emesis; and he must also have experienced the difficulty and uncertainty of obtaining it by any of the emetic substances in common use. Take, for instance, a case of spasmodic and intermittent croup. The patient is suddenly seized, for the most part in the night, with croupy cough and spasmodic breathing, which symptoms continue, attended with great anxiety and distress, for several hours, and then pass off to return on the succeeding night with redoubled violence. In many instances, the intermissions of all morbid phenomena have been so imperfect, as to lull the friends of the little sufferer into a fatal security, until, by the accumulated power of repeated paroxysms, the patient is overwhelmed with disease, almost beyond the hope of recovery; when for the first time, medical aid is called for. A single case will better illustrate the difficulties the physician has to encounter, than any general description.

I was called, in the night of Feb. 5, 1845, to see Miss C., aged about 12 years. In the three preceding nights, at about the same hour, she had been seized with paroxysms, similar to the one in which I found her—each one continuing several hours, each of progressively increased severity, and each leaving her able to be about, during the day, with respiration so easy as to excite no alarm, and only a slight croupy cough. I found her with breathing extremely quick laborious, and stridulous, the chest heaving with convulsive throes, the countenance livid, the eyes wild and distracted, extreme jactitation, the pulse thread-like, vaccilating, and too rapid to be counted, the extremities, up to the body, of an icy coldness, in short, the patient appeared like one in the last agonies. Strong rubefacients were immediately applied to the whole surface, sinapisms to the spine and extremities, and heated blankets to the lower limbs. As soon as she could swallow—for the deglutition was extremely difficult—five grains of the turpeth mineral were given at once, and followed with the free use of mustard whey; this not vomiting her, after fifteen minutes I gave her five grains more, immediately upon swallowing which, vomiting commenced, and continued smartly for more than an hour. During this operation, and for some hours after, a free use was made of a decoction of seneka, as a stimulant diaphoretic. Her respiration began to improve immediately on vomiting, and, at the end of about four hours, it was comparatively good; the skin open, natural warmth restored, and the circulation equalized. The patient was then put upon the use of the following powder:

℞. Pulv. ipecac. comp., ℈j.

Hydrarg. chlorid. mit., ℈j.

Camphor. pulv., grs. xij.

M. ft. Chart. No. vj.

One to be taken every four hours; a free use to be made of the decoction of seneka in the intervals, and the bowels to be moved after 24 hours.

The emetic did not produce purgation. No paroxysm of difficult breathing returned. In forty-eight hours, my attendance ceased. I know of no other emetic substance that would have effectually met such a case. Ipecac., sulphate of zinc, &c., would have proved too feeble at best. Tartarized antimony, would, I conceive, have been too hazardous, from its nauseating and sedative effect, setting aside the uncertainty of its producing emesis in any reasonable dose, and the almost certainty of its producing exhausting catharsis. I repeat, then, that in this form and condition of croup, my experience furnishes me with no substitute for the turpeth mineral, as an emetic.

In the more inflammatory and less paroxysmal forms of this disease, with decided arterial excitement, and hot skin, from the commencement, tartarized antimony as a nauseant and emetic, may be, and undoubtedly is, with certain limitations, preferable. Still, I must say, that in all stages and conditions of this disease, where the equalizing and revellent effect of emesis is alone desired, I have never regretted having used the turpeth mineral, in preference to all other emetics. These remarks might easily be extended to the use of this remedy in some other forms of disease, and especially in some of the anginose affections. The hints above given will, however, enable the experienced physician to determine its applicability to such conditions, and I am warned to bring to a close these remarks, which you may find already too prolix.

It remains, then, only to say a few words upon the peculiarity of this substance as an emetic, and upon some of the objections urged against it. In the first place, the promptness and certainty of its operation belong to no other substance within my knowledge. It has seldom, if ever, failed to vomit efficiently, when administered in a proper dose, in any of the various conditions of the stomach, and of the system, in which I have given it. It usually acts in ten or fifteen minutes, and the dose should be repeated at those intervals, if the first fail, which rarely happens. In efficiency and revellent power, it is not, perhaps, quite equal to the tartarized antimony; it is, however, vastly superior, in these respects, to ipecac. or any other substance known to me. In safety, it is generally superior to antimony. Its emetic operation usually continues from an hour to an hour and a half, accompanied and followed by none of the distressing nausea, prostration, and depletion of antimony; but, on the contrary, leaving the patient with the invigorated feeling arising from equalized warmth and circulation. In its emetic operation, it has saldom, never in my recollection, been accompanied or followed by catharsis. I have never known it to be violent, nor otherwise than entirely safe in its operation, although I have given it in much larger doses than are usually directed; nor have I ever seen salivation follow its use as an emetic. So safe do I consider it, that in urgent cases I have not hesitated to put my patient under its full emetic operation, two or three times within 24 hours; nor have I seen ill consequences result from such practice. I am inclined to think that the dose should be somewhat larger than is usually recommended. From two to three grains may be given to a child two years old, and repeated in ten or fifteen minutes, until emesis is produced. If the first dose fails, the second usually acts as soon as it touches the stomach.

Dr. Morris had no doubt that every physician, of much practice, must have experienced the need of a safe and efficient emetic, under the circumstances stated in the communication of Dr. Hubbard, as a substitute for the more active and depressing articles, in general use.

In a case of croup, some years ago, in which Dr. Meigs was in consultation, where tartar emetic had been carried as far as was deemed prudent, Dr. Meigs proposed the use of powdered alum, which answered an admirable purpose. Dr. Morris has since, repeatedly, used this article under similar circumstances, and has found it very rapid and efficient. It is an old remedy, but the suggestion for its use, under the circumstances referred to, came to him through Dr. Meigs, and he would confidently recommend it to the fellows.

Dr. Bell mistrusted the power of emetics, on the ground of their emetic effects alone, in the cases referred to in the communication of Dr. Hubbard. He considers croup to be an inflammatory disease, and preferred to place his reliance on depletion, and the antiphlogistic powers of tartar emetic. He considers the enfeebling effects of these remedies less to be dreaded, than the inflammation and morbid accumulations, which are too apt to follow a less energetic practice. The fact of the paroxysmal character of croup, is apt to deceive physicians into the idea, that no inflammation exists, whereas. Dr. B. believes that inflammations are very apt to assume a paroxysmal form, especially in croup. Dissections of those dying of this disease, manifest the pre-existance of inflammation of the larynx and trachea; and the same inference may be drawn from the repetition of the symptoms, after an apparent subsidence. One attack may occur to-night, and be relieved by morning, by mild means, whereas, on the following night, a still more violent paroxysm may unexpectedly occur, which shall destroy the patient, showing the probable continued existence of the disease, in a latent state. Dr. B. thought that the effects of Coxe's Hive Syrup, when used as a domestic remedy, should not be confounded with those of tartar emetic, cautiously prescribed, and its effects carefully watched by the physician. It must be remembered that the former is a compound article, containg, amongst other things, squills, which is itself a very depressing article—besides, the injudicious use of this compound by parents and nurses, is no argument against its employment, under proper restrictions. To return to the effects of tartarized antimony, in the disease under consideration. Dr. B. was unable to find any substitute for it—any article having less power, would, in his opinion, be inadequate to the effect desired. Dr. B. did not know that he had ever treated a case of croup (and he has seen many) without it, and he had never witnessed any alarming effects from it, except in one instance.

That was in a little girl, five or six years of age, who had an attack of croup in the night, which was relieved by morning, under the use of tart. emetic., administered under Dr. B.'s immediate notice. A repetition of the medicine was directed, in the event of a return of the croupal cough, and the mother, misunderstanding the directions, gave it repeatedly, and at the short intervals, as had been done during the height of the paroxysm. The prostration which ensued was so alarming as to induce the attendants to send for the Doctor; but in a short time, and though the patient was in a critical condition, she was effectually aroused by cordials and stimulants.

Dr. Bell agreed with Dr. Morris, as to the exceeding rarity of spasmodic croup, and when this form does occur. he knows of no remedy more likely to relieve it, than the tartar emetic.

Dr. Bell believes that the confidence reposed by American physicians in the use of the lancet, tartar emetic, and calomel in croup, was the ground of their superior success in the treatment of this disease; whereas, our trans-atlantic brethren, who are fearful of the use of these heroic remedies, do not enjoy, and probably from this very reason, the same success.

Dr. Bell, therefore, prefers to meet the disease by a frank, decided treatment in the outset, and, instead of simply relying on vomiting and anti-spasmodics, he prefers tartar emetic and the lancet, with a view to their depressing effect.

